# Supplementary material for: Causal Relationship between Aging and Anorexia Nervosa: A White-Matter-Microstructure-Mediated Mendelian Randomization Analysis
Source: Biomedicines. 2024 Aug 16;12(8):1874. doi: 10.3390/biomedicines12081874 (PMC11351342; doi:10.3390/biomedicines12081874)
Supplement: Supplementary file 1 [file biomedicines-12-01874-s001.zip › Supplementary Figure/Figure S4/Singe SNP plot of MR analysis for TL to ubm-b-1793.pdf]

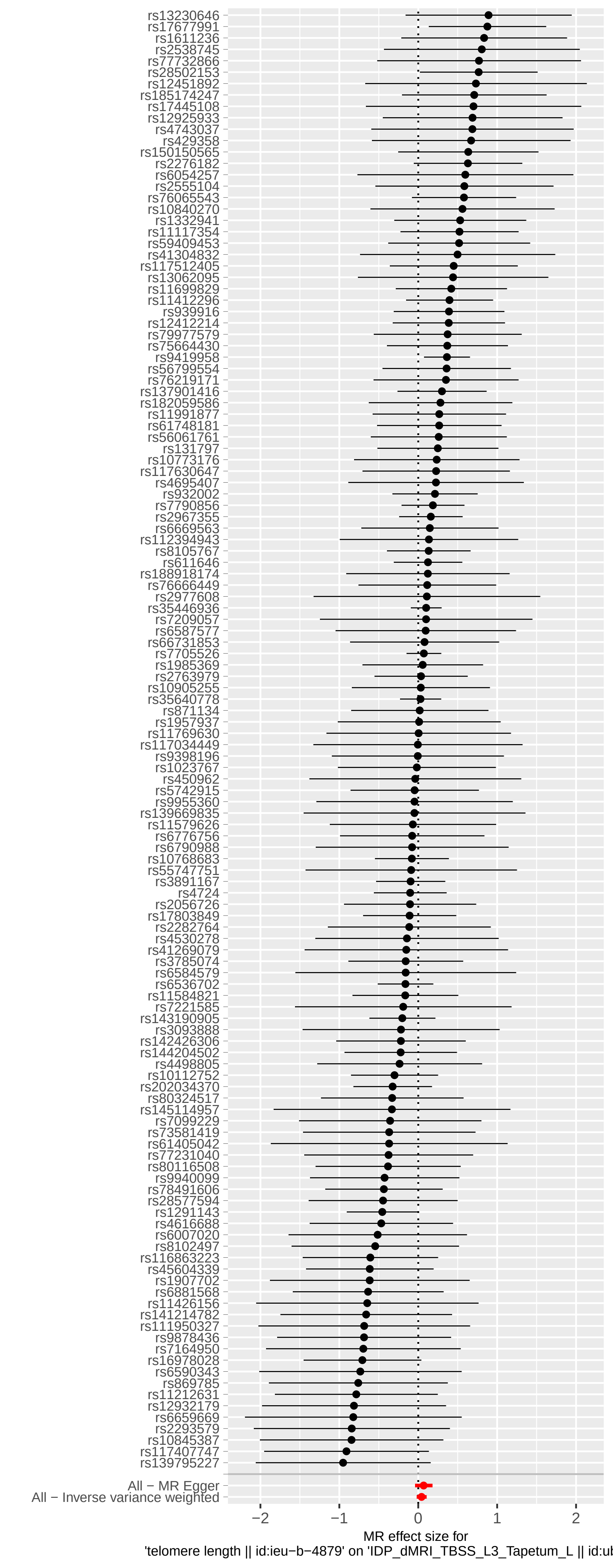

rs13230646
rs17677991
rs1611236
rs2538745
rs77732866
rs28502153
rs12451892
rs185174247
rs17445108
rs12925933
rs4743037
rs429358
rs150150565
rs2276182
rs6054257
rs2555104
rs76065543
rs10840270
rs1332941
rs11117354
rs59409453
rs41304832
rs117512405
rs13062095
rs11699829
rs11412296
rs939916
rs12412214
rs79977579
rs75664430
rs9419958
rs56799554
rs76219171
rs137901416
rs182059586
rs11991877
rs61748181
rs56061761
rs131797
rs10773176
rs117630647
rs4695407
rs932002
rs7790856
rs2967355
rs6669563
rs112394943
rs8105767
rs611646
rs188918174
rs76666449
rs2977608
rs35446936
rs7209057
rs6587577
rs66731853
rs7705526
rs1985369
rs2763979
rs10905255
rs35640778
rs871134
rs1957937
rs11769630
rs117034449
rs9398196
rs1023767
rs450962
rs5742915
rs9955360
rs139669835
rs11579626
rs6776756
rs6790988
rs10768683
rs55747751
rs3891167
rs4724
rs2056726
rs17803849
rs2282764
rs4530278
rs41269079
rs3785074
rs6584579
rs6536702
rs11584821
rs7221585
rs143190905
rs3093888
rs142426306
rs144204502
rs4498805
rs10112752
rs202034370
rs80324517
rs145114957
rs7099229
rs73581419
rs61405042
rs77231040
rs80116508
rs9940099
rs78491606
rs28577594
rs1291143
rs4616688
rs6007020
rs8102497
rs116863223
rs45604339
rs1907702
rs6881568
rs11426156
rs141214782
rs111950327
rs9878436
rs7164950
rs16978028
rs6590343
rs869785
rs11212631
rs12932179
rs6659669
rs2293579
rs10845387
rs117407747
rs139795227
All − MR Egger
All − Inverse variance weighted
−2
−1
0
1
2
MR effect size for
'telomere length || id:ieu−b−4879' on 'IDP_dMRI_TBSS_L3_Tapetum_L || id:ub
